# Association of Neuropathic Pain Symptoms with Sensitization Related Symptomatology in Women with Fibromyalgia

**DOI:** 10.3390/biomedicines10030612

**Published:** 2022-03-06

**Authors:** Edurne Úbeda-D’Ocasar, Juan Antonio Valera-Calero, Gracia María Gallego-Sendarrubias, César Fernández-de-las-Peñas, José Luis Arias-Buría, Matilde Morales-Cabezas, Lars Arendt-Nielsen, Margarita Cigarán-Méndez

**Affiliations:** 1 Department of Physiotherapy, Faculty of Health, Universidad Camilo José Cela, 28692 Villanueva de la Cañada, Spain; eubeda@ucjc.edu (E.Ú.-D.); gmgallego@ucjc.edu (G.M.G.-S.); 2 VALTRADOFI Research Group, Department of Physiotherapy, Faculty of Health, Universidad Camilo José Cela, 28692 Villanueva de la Cañada, Spain; 3 Department of Physical Therapy, Occupational Therapy, Rehabilitation and Physical Medicine, Universidad Rey Juan Carlos, 28922 Alcorcón, Spain; joseluis.arias@urjc.es (J.L.A.-B.); matilde.morales@urjc.es (M.M.-C.); 4 Center for Neuroplasticity and Pain (CNAP), Sanse-Motorisk Interaktion (SMI), Department of Health Science and Technology, Faculty of Medicine, Aalborg University, 9220 Aalborg, Denmark; lan@hst.aau.dk; 5 Department of Medical Gastroenterology, Mech-Sense, Aalborg University Hospital, 9000 Aalborg, Denmark; 6 Department of Psychology, Universidad Rey Juan Carlos, 28922 Alcorcón, Spain; margarita.cigaran@urjc.es

**Keywords:** fibromyalgia, neuropathic pain, chronic pain, sensitization, pressure threshold

## Abstract

We aimed to analyze potential correlations between S-LANSS and PainDETECT with proxies for pain sensitization, e.g., the Central Sensitization Inventory (CSI) and pressure pain hyperalgesia (construct validity), pain-related or psychological variables (concurrent validity) in women with fibromyalgia (FMS). One-hundred-and-twenty-six females with FMS completed demographic, pain-related variables, psychological, and sensitization outcomes as well as the S-LANSS and the PainDETECT questionnaires. S-LANSS was positively associated with BMI (r = 0.206), pain intensity (r = 0.206 to 0.298) and CSI score (r = 0.336) and negatively associated with all PPTs (r = −0.180 to −0.336). PainDETECT was negatively associated with age (r = −0.272) and all PPTs (r = −0.226 to −0.378) and positively correlated with pain intensity (r = 0.258 to 0.439), CSI (r = 0.538), anxiety (r = 0.246) and depression (r = 0.258). 51.4% of the S-LANSS was explained by PainDETECT (45.3%), posterior iliac PPT (0.2%) and mastoid PPT (5.9%), whereas the 56.4% of PainDETECT was explained by S-LANSS (43.4%), CSI (10.4%), and pain intensity (2.6%). This study found good convergent association between S-LANSS and PainDETECT in women with FMS. Additionally, S-LANSS was associated with PPTs whereas PainDETECT was associated with pain intensity and CSI, suggesting that both questionnaires assess different spectrums of the neuropathic and pain sensitization components of a condition and hence provide synergistic information.

## 1. Introduction

Fibromyalgia syndrome (FMS) affects 0.2–6.6% of the global population [1] and courses with widespread pain, sleep disturbances, fatigue, decreased health-related quality of life and function, and hyperalgesia and allodynic responses [2]. Although signs and symptoms of FMS are well described, underlying mechanisms are poorly understood and currently it is mainly considered a central sensitivity syndrome [3]. Evidence suggests the presence of abnormalities in ascending and descending central pain pathways and also changes at the level of neurotransmitters, explaining the augmented pain responses [4,5].

Since structural lesions in the somatosensory system are difficult to identify in patients with FMS, this condition is excluded from the definition of neuropathic pain [6], although common clinical symptoms found in FMS, e.g., tingling, numbness, burning paresthesia, hyperalgesia and allodynia, are compatible with a neuropathic pain phenotype. In such a scenario, FMS was originally classified as a nociceptive pain [7]. Additionally, FMS has been recently classified as a nociplastic pain condition, i.e., “pain arising from altered nociception without evidence of actual or threatened tissue damage causing the activation of peripheral nociceptors or evidence for disease or lesion of the somatosensory system causing the pain” [8].

Nevertheless, there is also evidence supporting the presence of small fiber neuropathy in FMS patients, compatible with the presence of neuropathic pain [9,10,11,12,13]. Self-reported questionnaires are fast and helpful tools for identifying individuals with neuropathic pain during clinical practice in a wide range of conditions. Two of the most commonly used questionnaires are the Self-Report Leeds Assessment of Neuropathic Symptoms (S-LANSS) [14] and PainDETECT [15]. Both questionnaires have shown an acceptable sensitivity (74% and 85%, respectively) and specificity (76% and 80%, respectively) when compared with nociceptive pain conditions [13].

Some studies have used the LANSS or PainDETECT questionnaires to investigate whether FMS exhibits a neuropathic pain component [13,16,17,18]. These studies also assessed the correlation between PainDETECT and S-LANSS with other features such as functional impact, tender point count, and clinical pain features of FMS [13,16,17,18]. In fact, the presence of tender points and widespread pain in FMS has been also associated with the presence of neuropathic pain symptoms as assessed with the S-LANSS [19]. To the best of the authors’ knowledge, no study has previously investigated if the presence of neuropathic symptoms assessed with the S-LANSS and PainDETECT questionnaires are associated to the presence of sensitization associated symptoms. Additionally, only the study by Kösehasanoğullari et al. included both questionnaires [13], therefore, the convergent validity between the S-LANSS and PainDETECT in FMS is still unclear and their applications is fibromyalgia are still not fully explored. Accordingly, the main aim of this study was to analyze the correlations between S-LANSS and PainDETECT with proxied for pain sensitization, e.g., the Central Sensitization Inventory (CSI) and widespread pressure pain hyperalgesia (construct validity), pain-related and psychological variables (concurrent validity) in women with FMS. A secondary aim was to conduct a linear regression model to explain the variance and identify those variables contributing to either S-LANSS or PainDETECT scores to explore possible complementary information provided by the two assessments.

## 2. Materials and Methods

### 2.1. Study Design

An observational cross-sectional study following the Strengthening the Reporting of Observational studies in Epidemiology (STROBE) guidelines [20] was conducted. The study design was approved by the Institutional Ethics Committee of Camilo José Cela University (UCJC 20 October 2020) and Universidad Rey Juan Carlos (URJC 30 August 2020). All participants signed written informed consent prior to their inclusion in the study.

### 2.2. Participants

Women with a medical diagnosis of FMS [21,22] aged between 20 and 70 years recruited at AFINSYFACRO Fibromyalgia Association located in Madrid (Spain) were screened for eligibility criteria. Volunteers were recruited from those responding to local announcements. Exclusion criteria included previous whiplash injury, previous surgery, neuropathic conditions (e.g., radiculopathy or myelopathy), comorbid medical conditions (e.g., tumor), or regular medication use affecting muscle tone or pain perception except non-steroidal anti-inflammatory drugs (NSAIDs).

Sociodemographic and clinical data were self-reported by the patients. Participants rated, on different 11-points numerical point rate scales (NPRS; 0: no pain; 10: maximum pain), their mean pain intensity at rest, the worst pain intensity at rest, and the level of pain experienced during daily living activities [23].

### 2.3. Neuropathic Pain Assessment: S-LANSS and PainDETECT

The S-LANSS questionnaire uses a binary response where individuals confirm whether they experience symptoms to classify them into a predominantly or non-predominantly neuropathic origin [14]. It has good sensitivity, internal consistency and validity [14]. The score ranges from 0 to 24, where those patients obtaining ≥12 points are susceptible of neuropathic pain [14].

The PainDETECT questionnaire is a self-reported questionnaire for measuring the presence of a neuropathic pain with has shown high sensitivity (85%), specificity (80%) and positive predictive accuracy (83%) [15]. This questionnaire consists of 9 items (seven pain-symptom items, one pain-course, and one pain-irradiation) completed into different scales. The total score ranges from 0 to 38, where higher scores indicate higher levels of neuropathic pain. PainDETECT assesses if a neuropathic pain component if unlikely (<12 points), ambiguous (12–18 points), or likely (>18 points) [15].

### 2.4. Central Sensitization Inventory

The CSI is a self-reported questionnaire assessing symptoms associated with central sensitization although the causal relation cannot be proven. It assesses 25 health-related symptoms common to assumed central sensitization with a 5-point Likert scale resulting in a score ranging from 0 to 100, where >40 points suggest hyper-excitability of the central nervous system [24]. This questionnaire also has a second part analyzing if patients have previously been diagnosed with several specific diagnoses, e.g., tension-type headache/migraines, fibromyalgia, irritable bowel syndrome, restless leg syndrome, temporomandibular joint disorder, chronic fatigue syndrome, and multiple chemical sensitivities, but this part is not included in the total score [24].

### 2.5. Pressure Pain Thresholds

The minimal amount of pressure needed to first change the sensation of pressure to pain, i.e., PPT, was bilaterally assessed with an electronic algometer (Somedic^®^, Sollentuna, Sweden) over the mastoid process, the upper trapezius muscle, the lateral epicondyle, the second metacarpal, the posterosuperior iliac spine, the greater trochanter, pes anserine, and tibialis anterior muscle since those have shown to be the most relevant locations to be assessed in FMS populations [25]. Pressure was applied at a rate of approx. 30 kPa/s (1 cm^2^ probe area) on each point. Participants were trained to press the “stop” button as soon as they felt the sensation first change from pressure to pain. The mean of 3 trials on each point was calculated and finally analyzed [25]. A resting period of 30 s was applied between each assessed point to avoid temporal summation [26]. This testing procedure has shown good reliability (ICC ≥ 0.88) in patients with FMS [27]. Since no side-to-side differences were found (independent Student’s *t*-tests), the mean of both sides was used in the analysis.

### 2.6. Hospital Anxiety and Depression Scale

Depression and anxiety were assessed with the HADS, a self-reported questionnaire consisting of 7 items assessing anxiety (HADS-A, 0–21 points) and 7 items assessing depressive levels (HADS-D, 0–21 points) on different 4-point Likert scales. Lower scores are associated with lower depressive and anxiety levels [28].

### 2.7. Sample Size Determination

An adequate sample size for prediction models was based on a range of 10 to 15 subjects per potential predictor variable [29], with no more than 5 predictors (as recommended by Beneciuk et al. [30]) within the model. Accordingly, for five potential predictor variables, a minimum of 75 participants would be required.

### 2.8. Statistical Analysis

All statistical analyses were conducted using the SPSS software v.27 for Mac OS (Armonk, NY, USA). Descriptive analyses (means and standard deviations -SD-) were used to describe the sample. The Kolmogorov-Smirnov test revealed that all quantitative data exhibited a normal distribution. A multiple linear regression analysis was used to determine which variables could explain the variance of either S-LANSS or PainDETECT. The following baseline variables were considered as potential predictors and included in the model: age, height, weight, body mass index, years with pain, years with diagnosis, pain intensity, PPTs, depression, anxiety, and CSI.

First, correlations between predictors and the dependent variables were assessed by using Pearson correlation coefficients (r). In addition, correlation coefficients were used to identify multicollinearity and shared variance between the variables (defined as r > 0.8). All statistically significant variables associated with S-LANSS and PainDETECT in the bivariate correlation analysis were included into two independent stepwise multiple linear regression models (hierarchical regression analysis) to assess the independent variables that contributed significantly to the variance of each dependent variables, except variables showing multicollinearity. The significance criterion of the critical F value for entry into the regression equation was set at *p* < 0.05. Changes in adjusted *R*^2^ were reported after each step of the regression model to determine the association of the additional variables.

## 3. Results

One-hundred-and-forty (n = 140) women with FMS were screened for eligible criteria. Fourteen (10%) were excluded due to previous surgery (n = 8), previous whiplash (n = 4), and pregnancy (n = 2). Finally, 126 women (mean age: 52.5 ± 11.0 years old) were satisfied all criteria and agreed to participate. Table 1 details sociodemographic, clinical and psychological characteristics of the total sample.

### 3.1. Bivariate Correlation Analysis

Bivariate correlation analysis results are reported in Table 2**.** S-LANSS was negatively associated with all PPTs (r = −0.180 to −0.333) and positively associated with BMI (r = 0.206), pain intensity (r = 0.206 to 0.298) and CSI (r = 0.336). PainDETECT was negatively associated with age (r = −0.272) and PPTs (r = −0.226 to −0.378) and positively associated with pain intensity at rest (r = 0.258), pain during daily life activities (r = 0.258), CSI (r = 0.538), anxiety (r = 0.246), depression (r = 0.258), and S-LANSS (r = 0.672).

Thus, multiple significant correlations existed among PPT locations (r: 0.303 to 0.812). Therefore, as multicollinearity (defined as r > 0.8) was identified for PPT located at tibialis anterior muscle with PPTs at elbow and greater trochanter, accordingly, PPT at tibialis anterior muscle was excluded from regression analyses.

### 3.2. Multiple Regression Analysis

**Figure 1:**
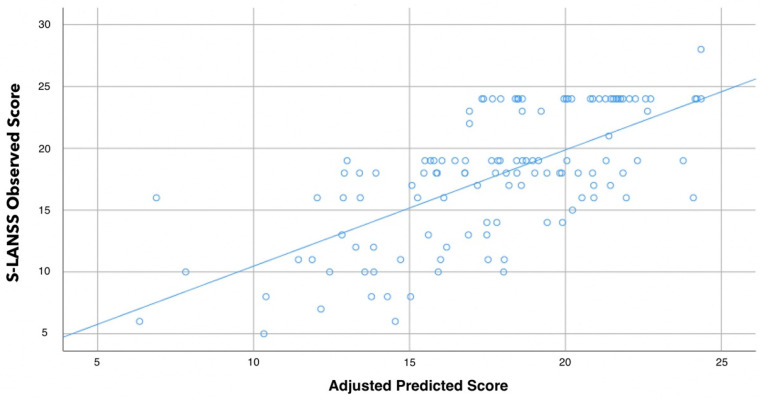
Scatter plot of the adjusted predicted score (r^2^ adjusted: 0.514) explaining the self-reported Leeds Assessment of Neuropathic Symptoms (S-LANSS) in women with fibromyalgia (n = 126). Note that some points can be overlapping.

The hierarchical stepwise regression analysis revealed that PainDETECT (contributing 45.3%), PPTs over posterior iliac crest (0.2%), and the mastoid process (5.9%) were significant predictors of S-LANSS and, when combined, they explained 51.4% of the variance (r^2^ adjusted: 0.514, Table 3, Figure 1).

**Figure 2:**
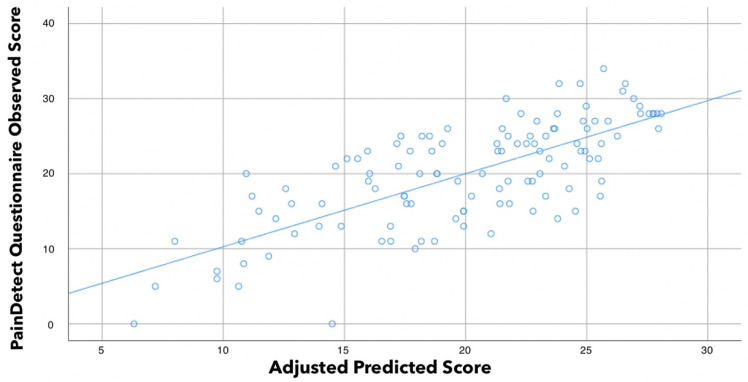
Scatter plot of the adjusted predicted score (r^2^ adjusted: 0.564) explaining the PainDETECT score in women with fibromyalgia (n = 126). Note that some points can be overlapping.

Similarly, stepwise regression analysis also revealed that S-LANSS (contributing 43.4%), CSI (10.4%), and pain intensity during daily living activities (2.6%) were significant predictors for PainDETECT and were able to explain 56.4% of accuracy (r^2^ adjusted: 0.564, Table 4, Figure 2).

## 4. Discussion

This is the first study assessing the association between neuropathic pain symptoms and sensitization associated symptomatology in women with FMS. We observed that the S-LANSS was associated with PPTs whereas PainDETECT was associated with pain intensity and CSI, suggesting that both variables could assess different aspects of the neuropathic pain spectrum. Further, we also found good convergent association between S-LANSS and PainDETECT, since each questionnaire explained almost 45% of the variance of the other one. Current results suggest that using both S-LANSS and PainDETECT in assessing FMS patients adds to the overall information achieved.

Giske et al. observed that identification of neuropathic pain symptoms in patients with widespread musculoskeletal pain is stable with time [31]. Previous studies have identified the presence of neuropathic pain symptoms in FMS [13,16,17,18]; however, the results are heterogeneous. Gauffin et al. reported the presence of neuropathic pain symptoms in 34% of their sample [17], whereas Amris et al. observed in almost 75% [18]. Both studies used the PainDETECT score (cut-off > 18 points). In our study, according to S-LANSS (cut-off ≥12 points) 20% (n = 26) of women with FMS exhibited neuropathic pain symptoms, whereas according to PainDETECT (cut-off > 18 points) the prevalence of neuropathic likely-symptoms was 38.8% (n = 49). Discrepancies between the studies could be related to the fact that FMS is a heterogeneous syndrome [32]. Additionally, it is also possible that S-LANSS and PainDETECT, although both evaluate the presence of neuropathic pain symptoms, may assess different components of the neuropathic pain spectrum. This assumption would be supported by the fact that those variables independently associated with each questionnaire on the regression analysis were different and specific for each. Nevertheless, we found a moderate association between S-LANSS and PainDETECT in the bivariate analysis and also a good convergence since each questionnaire explained almost 45% of the variance of the other one in the regression analysis, in agreement with a previous study reporting a stronger correlation between both questionnaires than with measures of pain severity in people with knee osteoarthritis [33]. Current and previous results suggest good convergent validity between both S-LANSS and PainDETECT.

Sensitivity changes are consistent in individuals with chronic pain conditions and sensitization [34,35]. Sensitization in FMS is characterized by generalized lowered PPTs and elevated widespread pain in response to different stimuli [3,4]. In fact, it has been suggested that widespread pressure hyperalgesia represent a general central amplification—but importantly PPTs can only be a proxy for such manifestations. We observed small to moderate associations between widespread PPTs and S-LANSS and PainDETECT questionnaires, suggesting that neuropathic pain symptomatology is associated with higher pressure pain hypersensitivity in our sample of women with FMS. In agreement with our results, Amris et al. also showed negative associations between PainDETECT and PPTs [18]. Similarly, a higher number of tender points, another manifestation of pain hyperalgesia, has been associated with the presence of neuropathic pain symptoms in individuals with FMS [19]. Current results would agree with the fact that people with peripheral nerve injury exhibit sensory loss but also mechanical hyperalgesia [36]. Similarly, the presence of neuropathic symptom in a musculoskeletal pain condition such as knee osteoarthritis also lead to higher pressure pain hyperalgesia [37]. However, only PPTs over the mastoid and posterior iliac crest were independently associated with S-LANSS after adjusting by all the variables in the regression model suggesting that the contribution of pressure pain sensitivity to neuropathic symptoms is minimal. In fact, PPTs were not independently associated with PainDETECT score. Current data suggest that pressure pain hyperalgesia and the presence of neuropathic symptoms represent two different dimensions associated to sensitization (peripheral or central). This hypothesis is further supported by the fact that sensitization associated symptoms, i.e., CSI score, was independently associated with PainDETECT in the current study.

We also found intensity of pain at rest and during daily living activities to be positively associated with S-LANSS and PainDETECT scores suggesting that the magnitude of the nociceptive driving is a relevant factor associated with the presence of neuropathic pain symptoms and sensitization [38]. Nevertheless, only the intensity of pain with daily living activities was an independent contributor to PainDETECT, but not S-LANSS, score after adjusting by all variables. Our results agree with previous studies reporting a correlation between PainDETECT and S-LANSS with clinical pain features in FMS [13,16,17,18]. In fact, Gauffin et al. reported an association between the mean and the worst pain intensity with the presence of neuropathic pain [17]. However, none of these studies conducted a regression analysis considering the influence of the remaining variables.

Finally, some limitations associated to the current study are recognized. First, only women were recruited. Therefore, current findings cannot be extrapolated to males with FMS. Second, we assessed depression and anxiety as psychological variables. It is possible that other cognitive variables, e.g., pain hypervigilance, catastrophism, or kinesiophobia could also contribute to the presence of neuropathic pain symptomatology in women with FMS. Third, analyzing further PPT locations could be of interest for better understanding of the association between neuropathic pain symptomatology and widespread pressure pain hyperalgesia in women with FMS. Fourth, we used the S-LANSS and the PainDETECT to assess the presence of neuropathic pain symptoms. Other questionnaires, such as the Douleur Neuropathique-4 items (DN4) [39] or Neuropathic Pain Symptoms Inventory (NPSI) [40] can provide different information. Finally, prospective studies assessing the longitudinal association between neuropathic pain and sensitization associated symptomatology in women with FMS could help to highlight the usefulness of current results.

## 5. Conclusions

The current study observed that the S-LANSS was mainly and independently associated with PPTs, whereas PainDETECT was associated with pain intensity and CSI, suggesting that both questionnaires could assess different aspects of neuropathic pain spectrum and thereby add synergistic information. In addition, a good convergent association between S-LANSS and PainDETECT was also found, since each questionnaire explained almost 45% of the variance of the other one in women with FMS.

## Figures and Tables

**Table 1:** Clinical data (mean ± SD) of the sample.

Baseline Variable	Female Patients with FMS (n = 126)
*Sociodemographic characteristics*	
Age (years)	52.0 ± 10.7
Height (m)	1.61 ± 0.06
Weight (kg)	71.4 ± 16.6
Body Mass Index (kg/cm^2^)	27.5 ± 6.2
*Clinical characteristics*	
Years with pain (years)	20.1 ± 15.3
Years with FMS (years)	10.2 ± 8.9
Pain intensity (NPRS, 0–10)	
Mean at rest	6.4 ± 1.7
Worst at rest	7.3 ± 2.2
During daily activities	8.1 ± 1.9
CSI (0–100)	70.7 ± 11.6
S-LANSS (0–24)	17.5 ± 5.5
PainDETECT (0–38)	19.9 ± 7.1
*Psychological characteristics*	
HADS-A (0–21)	11.4 ± 3.7
HADS-D (0–21)	10.0 ± 4.0
*Pressure Pain Thresholds*	
PPT Mastoid (kPa)	151.2 ± 90.7
PPT Upper Trapezius (kPa)	125.6 ± 60.4
PPT Elbow (kPa)	149.0 ± 87.1
PPT Hand (kPa)	120.2 ± 59.0
PPT Posterosuperior Iliac Spine (kPa)	233.9 ± 130.7
PPT Greater Trochanter (kPa)	257.7 ± 123.9
PPT Knee (kPa)	148.1 ± 107.1
PPT Tibialis Anterior (kPa)	187.0 ± 108.7

FMS: fibromyalgia syndrome; HADS: Hospital Anxiety and Depression Scale; PPT: pressure pain threshold; S-LANSS: self-reported version of the Leeds Assessment of Neuropathic Symptoms and Signs.

**Table 2:** Pearson-product moment correlation matrix between sociodemographic, psychological, neuro-physiological and clinical characteristics.

	1	2	3	4	5	6	7	8	9	10	11	12	13	14	15	16	17	18	19
1. Age																			
2. BMI	n.s.																		
3. Years with pain	0.566 **	n.s.																	
4. Years with FMS	0.598 **	n.s.	0.615 **																
5. Mean pain at rest	n.s.	n.s.	n.s.	n.s.															
6. Worst pain at rest	n.s.	n.s.	n.s.	n.s.	0.427 **														
7. PDDA	n.s.	n.s.	n.s.	n.s.	0.302 **	n.s.													
8. Mastoid PPT	0.201 *	n.s.	n.s.	n.s.	n.s.	n.s.	n.s.												
9. Trapezius PPT	n.s.	n.s.	n.s.	n.s.	n.s.	n.s.	n.s.	0.399 **											
10. Elbow PPT	0.244 **	n.s.	n.s.	n.s.	n.s.	n.s.	n.s.	0.535 **	0.580 **										
11. Hand PPT	0.192 *	n.s.	n.s.	n.s.	n.s.	n.s.	n.s.	0.519 **	0.564 **	0.726 **									
12. Posterior Iliac PPT	0.195 *	n.s.	n.s.	n.s.	n.s.	n.s.	n.s.	0.502 **	0.565 **	0.756 **	0.563 **								
13. Trochanter PPT	n.s.	n.s.	n.s.	n.s.	n.s.	n.s.	−0.265 **	0.439 **	0.625 **	0.711 **	0.609 **	0.658 **							
14. Knee PPT	n.s.	n.s.	n.s.	n.s.	n.s.	n.s.	n.s.	0.303 **	0.516 **	0.470 **	0.461 **	0.458 **	0.523 **						
15. Tibialis PPT	n.s.	n.s.	n.s.	n.s.	n.s.	n.s.	−0.195 *	0.492 **	0.660 **	0.804 **	0.714 **	0.712 **	0.812 **	0.601 **					
16. CSI	−0.262 **	n.s.	n.s.	n.s.	0.305 **	0.249 **	0.398 **	−0.371 **	−0.263 **	−0.341 **	−0.221 *	−0.372 **	−0.418 **	−0.228 *	−0.332 **				
17. HADS-A	−0.179 *	n.s.	n.s.	−0.187 *	0.198 *	0.254 **	n.s.	−0.223 *	n.s.	n.s.	n.s.	n.s.	n.s.	n.s.	n.s.	0.541 **			
18. HADS-D	−0.181 *	n.s.	−0.299 **	−0.197 *	n.s.	n.s.	0.186 *	−0.230 *	−0.250 **	−0.245 **	−0.177 *	−0.189 *	−0.212 *	n.s.	−0.247 **	0.415 **	0.566 **		
19. S-LANSS	n.s.	0.206 *	n.s.	n.s.	0.237 **	0.206 *	0.298 **	n.s.	−0.187 *	−0.305 **	−0.180 *	−0.333 **	−0.234 **	−0.208 *	−0.239 **	0.336 **	n.s.	n.s.	
20. PainDetect	−0.272 **	n.s.	n.s.	n.s.	0.258 **	n.s.	0.439 **	−0.279 **	−0.271 **	−0.331 **	−0.280 **	−0.378 **	−0.352 **	−0.226 *	−0.343 **	0.538 **	0.246 **	0.258 **	0.672 **

BMI: body mass index; CSI: Central Sensitization Inventory; FMS: fibromyalgia syndrome; HADS: Hospital Anxiety and Depression Scale; PDDA: pain during daily activities; PPT: pressure pain threshold; S-LANSS: self-reported version of the Leeds Assessment of Neuropathic Symptoms and Signs. * *p* < 0.05; ** *p* < 0.01.

**Table 3:** Summary of the stepwise regression analyses to determine predictors of S-LANSS.

	Predictor Outcome	Β	SE B	95% CI	β	t	*p*
S-LANSS	Step 1						
	PainDETECT	0.519	0.051	0.418; 0.621	0.677	10.147	<0.001
	Step 2						
	PainDETECT	0.493	0.055	0.384; 0.603	0.643	8.928	0
	Posterior Iliac PPT	−0.004	0.003	−0.010; 0.002	−0.088	−1.227	0.222
	Step 3						
	PainDETECT	0.514	0.053	0.408; 0.619	0.669	9.656	<0.001
	Posterior Iliac PPT	−0.009	0.003	−0.015; −0.002	−0.208	−2.701	0.007
	Mastoid PPT	0.016	0.004	0.007; 0.024	0.258	3.485	0.001

CI: Confidence Interval; PPT: pressure pain thresholds; S-LANSS: self-reported version of the Leeds Assessment of Neuropathic Symptoms and Signs; SE: Standard Error. R^2^ adj. = 0.453 for step 1, R^2^ adj. = 0.455 for step 2, R^2^ adj. = 0.514 for step 3.

**Table 4:** Summary of the stepwise regression analyses to determine predictors of PainDETECT.

	Predictor Outcome	Β	SE B	95% CI	β	t	*p*
PainDETECT	Step 1						
	S-LANSS	0.891	0.094	0.704; 1.078	0.663	9.444	<0.001
	Step 2						
	S-LANSS	0.717	0.093	0.533; 0.901	0.533	7.723	<0.001
	CSI	0.203	0.041	0.121; 0.284	0.341	4.936	<0.001
	Step 3						
	S-LANSS	0.677	0.092	0.494; 0.859	0.503	7.348	<0.001
	CSI	0.168	0.042	0.084; 0.252	0.282	3.959	<0.001
	PDDA	0.662	0.258	0.150; 1.174	0.177	2.561	0.012

CI: Confidence Interval; CSI: Central Sensitization Inventory; PDDA: pain during daily activities; SE: Standard Error; S-LANSS: self-reported version of the Leeds Assessment of Neuropathic Symptoms and Signs. R^2^ adj. = 0.434 for step 1, R^2^ adj. = 0.538 for step 2, R^2^ adj. = 0.564 for step 3.

## Data Availability

All data relevant to the study are included in the article.
